# Integrative Analyses of Biochemical Properties and Transcriptome Reveal the Dynamic Changes in Leaf Senescence of Tobacco (*Nicotiana tabacum* L.)

**DOI:** 10.3389/fgene.2021.790167

**Published:** 2021-12-22

**Authors:** Binghui Zhang, Jiahan Yang, Gang Gu, Liao Jin, Chengliang Chen, Zhiqiang Lin, Jiangyu Song, Xiaofang Xie

**Affiliations:** ^1^ College of Life Sciences, Fujian Agriculture and Forestry University, Fuzhou, China; ^2^ Institute of Tobacco Science, Fujian Provincial Tobacco Company, Fuzhou, China; ^3^ Yanping Branch of Nanping Tobacco Company, Nanping, China; ^4^ Jianning Branch of Sanming Tobacco Company, Sanming, China; ^5^ Nanping Tobacco Company, Nanping, China

**Keywords:** *Nicotiana tabacum*, leaf senescence, transcriptome analysis, senescence-associated genes, co-expression network

## Abstract

Leaf senescence is an important process of growth and development in plant, and it is a programmed decline controlled by a series of genes. In this study, the biochemical properties and transcriptome at five maturity stages (M1∼M5) of tobacco leaves were analyzed to reveal the dynamic changes in leaf senescence of tobacco. A total of 722, 1,534, 3,723, and 6,933 genes were differentially expressed (DEG) between M1 and M2, M1 and M3, M1 and M4, and M1 and M5, respectively. Significant changes of nitrogen, sugars, and the DEGs related to metabolite accumulation were identified, suggesting the importance of energy metabolism during leaf senescence. Gene Ontology (GO) analysis found that DEGs were enriched in biosynthetic, metabolic, photosynthesis, and redox processes, and especially, the nitrogen metabolic pathways were closely related to the whole leaf senescence process (M1∼M5). All the DEGs were grouped into 12 expression profiles according to their distinct expression patterns based on Short Time-series Expression Miner (STEM) software analysis. Furthermore, Kyoto Encyclopedia of Genes and Genomes (KEGG) pathway analysis found that these DEGs were enriched in pathways of carbon metabolism, starch and sucrose metabolism, nitrogen metabolism, and photosynthesis among these expression profiles. A total of 30 core genes were examined by Weight Gene Co-expression Network Analysis (WGCNA), and they appeared to play a crucial role in the regulatory of tobacco senescence. Our results provided valuable information for further functional investigation of leaf senescence in plants.

## Introduction

Leaf senescence is an important trait that affects the biomass accumulation and nutritional value of agricultural crops. Common tobacco (*Nicotiana tabacum* L.) is regarded as an ideal model organism to investigate leaf senescence. The study of tobacco leaf senescence and its internal material transport provides an important platform for understanding tobacco plant growth and development (Gregersen et al., 2013). Leaf senescence is a complicated developmental process, which is regulated by internal genetic program and other environmental signals (Uzelac et al., 2016). Leaf senescence of tobacco is a complex process involving many molecular events along with physiological and biochemical changes, and these changes are driven by differential expression of thousands of genes under the control of highly regulated genetic procedures (Masclaux et al., 2000; Gregersen and Holm, 2007). As one of the regulated processes, the cells of leaves undergo orderly modifications in structure, metabolism, and gene expression, along with a series of degradations, including the chloroplast, photosynthetic proteins, and other macromolecules (Gan and Amasino, 1997; Lim et al., 2003; Schippers et al., 2015); the conversion of peroxisomes into glyoxysomes; and boosted production of ROS (Rogers, 2012), and thereby result in the chlorophyll content decrease and photosynthetic capacity decline (Lim et al., 2007; Lira et al., 2017). Usually, the formation of product organs is closely related to leaf senescence. To improve leaf quality and yield, a common cultivation practice of topping is used to regulate the nutrient distribution by changing the sink-to-source transition of the leaves and thus increase the dry matter accumulation in leaves (Weeks and Seltmann, 1986).

Leaf senescence is also driven by a series of genes called senescence-associated genes (SAGs), especially those encoding key regulators (Gan and Amasino, 1997; Chen et al., 2019). In many plants, the expression of some regulators involved in senescence such as transcription factors and translation elongation factors often exhibits upregulation before or in the process of leaf senescence (Liu et al., 2016). Previous report showed that phytohormones play crucial roles in influencing senescence (Khan et al., 2014). Some hormones such as cytokinin (CK), indoleacetic acid (IAA), and gibberellin (GA) may also play a positive role in delaying senescence (Gan, 2005; Lim et al., 2007), while some other hormones including ethylene (ET), abscisic acid (ABA), salicylic acid (SA), and jasmonic acid (JA) may involve in regulating the plant response to various stresses and accelerate plant senescence (Kong et al., 2013; Khan et al., 2014). It was found that there was a descent in cytokinin and an increase in ABA concentration at postharvest leaves (Miret et al., 2018).

To assess tobacco leaf maturity more appropriately, studies have been performed to evaluate the maturity using physiological and biochemical indexes, besides the conventional and morphological characters. However, these efforts still could not deeply reveal the molecular regulation mechanism of leaf senescence. With the availability of genome sequences and the rapid development of high-throughput tools since the early 2000s, RNA-seq technique provides a good means to obtain a comprehensive molecular insight of leaf senescence based on large-scale information of gene expression regulation. In recent years, progress has been made in understanding the molecular mechanism of leaf senescence in tobacco by using transcriptome analysis, especially the research on senescence regulatory factors and the signal transduction pathway. Zhao et al. (2018) investigated the gene expression patterns involved in premature senescence using the transcriptome analysis. To reveal nutrient remobilization events, Li et al. (2017) explored the expression changes of enzyme encoding the genes in corresponding metabolic pathways related to metabolite accumulation in different development stages of tobacco. In addition, RNA-seq was used for the study of gene expression patterns which participate in nicotine metabolism and carotenoid metabolism during senescence of tobacco (Wen et al., 2019).

To investigate the dynamics of nutrient accumulation and molecular events associated with nutrient remobilization in tobacco, we collected samples at different maturity stages of middle leaves and incorporated the information of morphological characters, physiological indexes, and transcriptome together for data analysis. The outcomes of the analysis enable us to decipher the association of functional categories of genes in different maturity leaves and thereby provide useful theoretical foundation at the molecular level for understanding leaf senescence.

## Materials and Methods

### Plant Materials and Sampling

*Nicotiana tabacum* cv. Cuibi 1 (CB-1) was used in this study, which is widely cultivated in south of China. Middle leaves (MLs) at the eighth to 10th positions were selected for this study. Leaves at five different maturity groups were collected at the same time and defined as M1, M2, M3, M4, and M5. The stages of maturities were judged by visible appearance, including the leaf yellowing rate, glandular trichome, and the angle between the stem and leaf (Ougham et al., 2008; Zhao et al., 2018). A total of six leaves were selected from each sample and divided into two subgroups along the boundary of the midrib (midvein), which were used for biochemical measurement and RNA extraction, respectively. Three biological replicates were collected for each maturity level.

### Chlorophyll Extraction and Quantification

Chlorophyll was extracted and quantified by following the method of He and Gan, (2002). Chlorophyll was extracted by using 95% ethanol. The supernatant was measured at 649 and 665 nm using a spectrophotometer (UV-1780, Shimadzu, Japan). Chlorophyll contents were calculated, and mean values were obtained based on three biological replicates.

### Chemical Composition Analysis

Total nitrogen, nicotine, starch, total soluble sugar, and reducing sugar content were assessed following the method of Chinese tobacco industry standard (YC/T 161-2002, YC/T 160-2002, YC/T 216-2007, and YC/T 159-2002) by using a continuous flow method. All chemical composition measuring methods were according to the description of the Chinese tobacco industry standard method (Wu et al., 2018).

### RNA Sequencing and Data Analysis

Total RNA was extracted using a total RNA isolation kit (PR2401, Bioteke Corporation, China). Agarose gel electrophoresis (1%) was used for measuring RNA quality. The concentration of each sample was examined using a NanoDrop-100 (Hangzhou Miu Instruments Co., Ltd., China). The RNA-seq library preparation was carried out following the method described by Li and Guo (2018). A total of 15 RNA samples (five senescence stages with three biological replicates, namely, M1-1, M1-2, M1-3, M2-1, M2-2, M2-3, M3-1, M3-2, M3-3, M4-1, M4-2, M4-3, M5-1, M5-2, and M5-3) were sequenced on Illumina HiSeq™ 2000 performed by Biomarker Technologies (http://www.biomarker.com.cn/, BioMarker, Beijing, China). Reads (100 bp in length, paired-end) were mapped to the tobacco reference genome (Edwards et al., 2017) (https://solgenomics.net/organism/Nicotiana _attenuata/genome). RNA-seq reads were assessed with FastQC version 0.10.1 (https://www.bioinformatics. babraham.ac.uk/projects/fastqc/) for quality control. Reads were mapped to a reference genome of tobacco genome sequences (Edwards et al., 2017) of the Sol Genomics Network database (https://solgenomics.net/organism/Nicotiana_attenuata/genome) using HISAT2 (Kim et al., 2015; https://ccb.jhu.edu/software/hisat2/index.shtml). To perform the gene expression analysis, the numbers of matched reads were normalized by the FPKM (fragments per kilobase per million) method (Trapnell et al., 2010). The DEGs were defined by the criteria of |log_2_(fold change)| ≥ 1.5 and false discovery rate (FDR) ≤ 0.05. All genes from the reference genome were annotated with BLAST2GO (Conesa et al., 2005; https://www . blast2go.com). The DEGs, including the upregulated or downregulated, were imported into the bioconductor package topGO (Alexa and Rahnenfuhrer, 2017; https://rdrr.io/bioc/topGO/) for gene ontology (GO) enrichment analysis. Short Time-series Expression Miner (STEM) software was applied to analyze the expression patterns of DEGs along with the increase in senescence degree of the tobacco leaf (Ernst and Bar-Joseph, 2006). With the log-normalized data option to run, with other parameters in default, the genes with similar expression patterns were clustered into the same profile with a *p*-value < 0.05, which was considered significantly enriched. To further understand the potential function of the DEGs in each profile obtained by the STEM analysis, the Kyoto Encyclopedia of Genes and Genomes (KEGG) pathway analysis was carried out using KAAS databases (https://www.genome.jp/tools/kaas/) (Moriya et al., 2007). The MapMan tool (http://MapMan.gabipd.org ) was used for a graphical overview of pathways involving the DEGs.

### Co-Expression Network Construction

To investigate the genes related to leaf senescence, gene expression profiles of tobacco leaves at five senescence stages were examined using weighted gene co-expression network analysis (WGCNA) in R package (Langfelder and Horvath, 2008). The DEGs obtained in pairwise comparisons across 15 transcriptomes with different senescence stages, including biological replicates, were used for network construction, while the genes with an FPKM <0.5 were filtered. Unigenes were hierarchically clustered according to the topological overlap matrix. The parameters for network construction were set as follows: power = 10, MEDissThres = 0.25, and nSelect = 400. The candidate genes related to leaf senescence were imported into the network tool Cytoscape (version 3.8.2; Shannon et al., 2003; https://cytoscape.org/) to generate a visualization of the interactors. The candidate hub genes were ranked by the Maximal Clique Centrality (MCC) method with Cytoscape software.

### Quantitative Real-Time PCR Analysis

To verify the quality of RNA-seq data, qRT-PCR was performed. cDNA was synthetized using a PrimeScript™ RT reagent kit with gDNA eraser (Takara, Japan) following the manufacturer’s instruction. The cDNA samples were then assayed by qRT-PCR using SYBR Premix Ex Taq (Takara) on the ABI 7500 fast Real-Time PCR System (Applied Biosystems, USA). The gene-specific primers used for qRT-PCR analysis are listed in Supplementary Table S1. The *actin* gene of tobacco was used as an internal control. Three biological replicates and three technical replicates were tested. The relative expression level of the detected gene was calculated using the 2^−ΔΔCt^ method (Livak and Schmittgen, 2001).

## Results

### Morphological Characteristics and Chemical Composition Content in Five Maturity Stages

**FIGURE 1:**
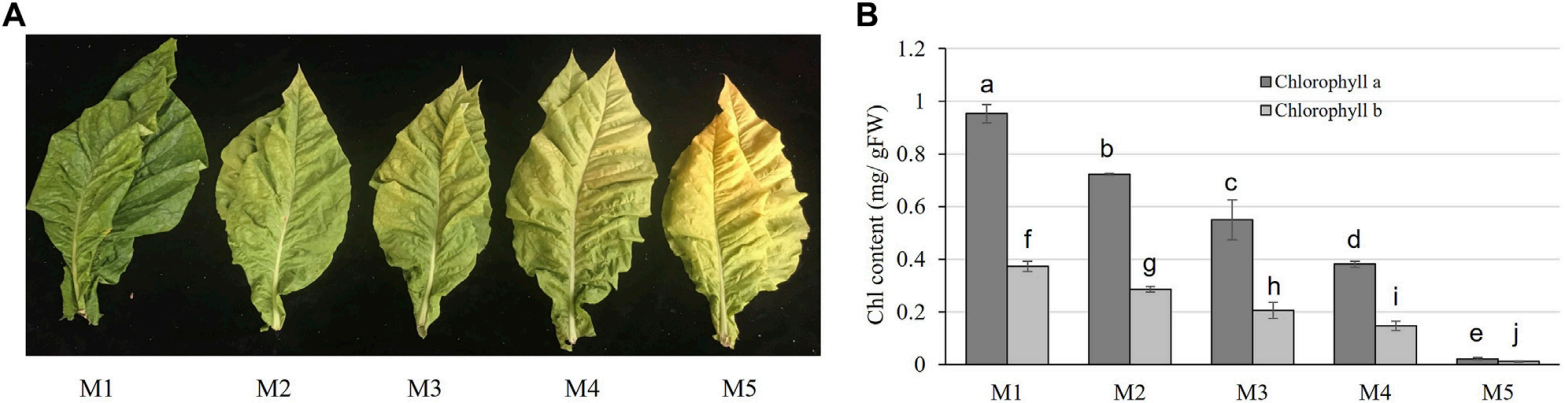
Character of tobacco leaves at five stages of maturity. **(A)** Appearance of tobacco leaves at five stages of maturity. **(B)** Chlorophyll (chl) content at the five stages of maturity. Error bars indicate the means ± SD (*n* = 3). Values with the different letter show significantly different according to the Duncan test (*p* > 0.05).

Five maturity stages of M1, M2, M3, M4, and M5 were collected based on the visible appearance. The yellowing rates of these five stages increased gradually with the increase in maturity (Figure 1A). Chlorophyll concentration was used to measure the photosynthetic capacity of leaves at five stages, and the significant declining tendency of the chlorophyll content was observed from M1 to M5 (with the increase in maturity degree) (Figure 1B).

**FIGURE 2:**
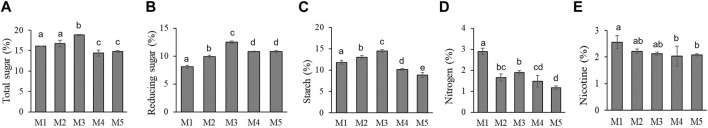
Chemical components at five maturity leaves of CB-1. **(A)** Total sugar. **(B)** Reducing sugar. **(C)** Starch. **(D)** Nitrogen. **(E)** Nicotine. Values with the same letter are not significantly different according to the Duncan test (*p* > 0.05).

To understand the effect of different maturity stages on the biochemical indexes, biochemical components of these leaves were measured (Figure 2). With the increase in the maturity level, the physiological indexes of tobacco leaves with different maturity degrees showed a coordinated collinearity. Among them, the highest contents of nicotine (2.56%) and nitrogen (2.9%) were found at M1 and then a decrease with increasing maturity. The content of total sugar, reducing sugar, and starch increased first and then dropped with increasing maturity, and the highest contents of total sugar, reducing sugar, and starch were found at M3 maturity.

### Gene Expression Data and Differentially Expressed Genes in Different Stages of Maturity

A total of 15 RNA samples were sequenced in this study. The data generated from RNA-seq of these 15 samples were in alignment to the tobacco reference genome after quality control. After quality control and alignment to the tobacco reference genome, we obtained a total of 779.08 M mapped reads, of which 672.14 M reads were uniquely mapped (Supplementary Table S2). The clean reads ranged from 45.31 to 62.42 Mb in different maturity stages, and more than 83% were uniquely mapped for each sample (Supplementary Table S2). The uniquely mapped reads were used for further gene expression analysis. The transcriptome data have been uploaded to the database of the NCBI Sequence Read Archive (http://trace.ncbi.nlm.nih.gov/Traces/sra) under the accession number PRJNA772550.

**FIGURE 3:**
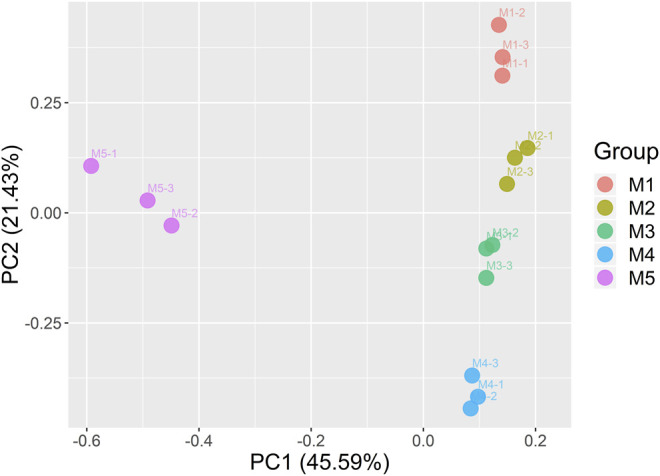
Principal component analysis of the genes identified from the 15 samples.

Principal component analysis (PCA) was further performed based on the aforementioned identified–expressed genes (Figure 3). The results showed that the expressed genes at the same maturity stage in three repeat samples could be aggregated well. The PCA on the gene expression based on different maturity stages indirectly proved, to some extent, the reliability of our RNA-seq data.

**FIGURE 4:**
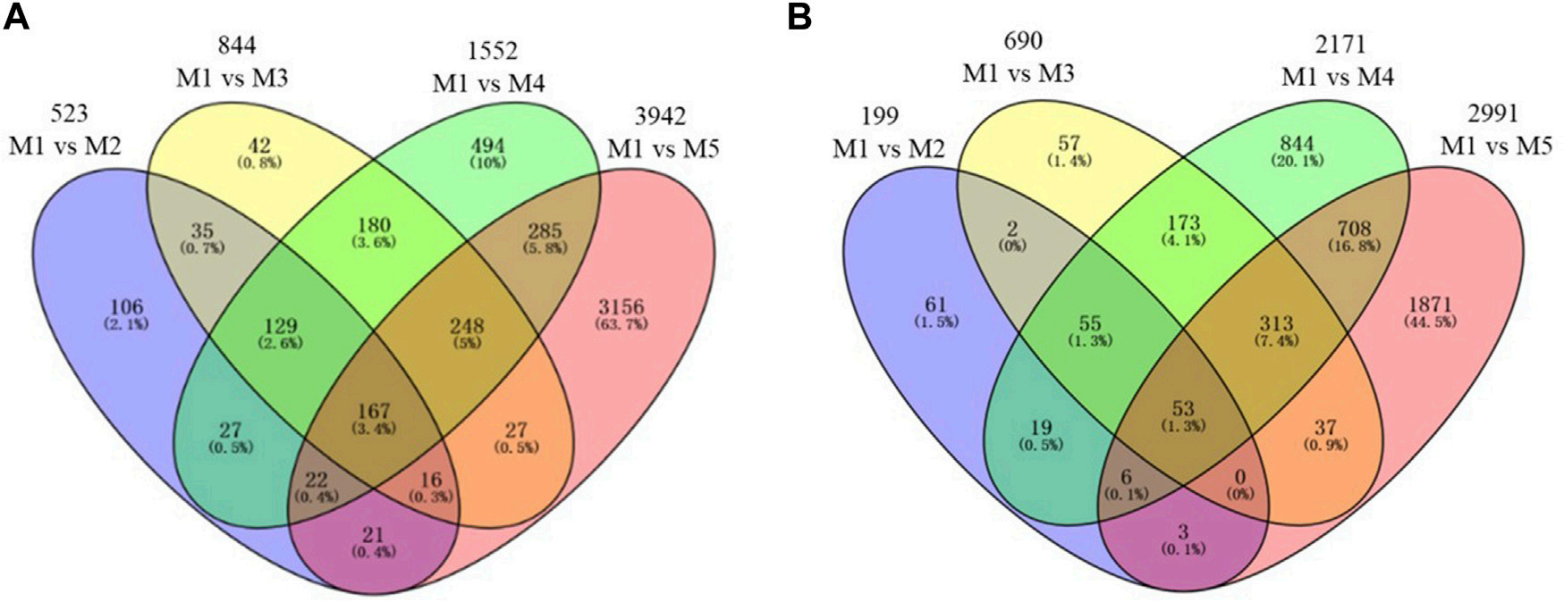
Venn diagram of differentially expressed genes (DEGs) detected by pair-wise comparison at five maturity stages. **(A)** Upregulated. **(B)** Downregulated.

To identify differentially expressed genes (DEGs), M1 was set as a reference stage and all other maturity stages were compared with M1, and four comparisons were analyzed including C1: M1 vs. M2; C2: M1 vs. M3; C3: M1 vs. M4, and C4: M1 vs. M5 (Figure 4). A total of 722 (523 + 199) DEGs were detected in C1 (Supplementary Table S3), among which ∼7/10 (523) genes were upregulated. In terms of C2 (Supplementary Table S4), a total of 1,534 DEGs were found with 844 upregulated genes and 690 downregulated genes. In the comparison to C3 (Supplementary Table S5), a total of 3,723 DEGs were detected including 1,552 upregulated and 2,171 downregulated genes. Moreover, there were more numbers of DEGs (6,933) in C4 than the other three comparisons, with 3,942 genes upregulated and 2,991 genes downregulated (Supplementary Table S6). The numbers of DEGs in C2, C3, and C4 increased quickly in comparing to C1 (∼2, ∼5, and, ∼10 times, respectively), with the increasing leaf senescence. The result indicated that numerous genes were involved in leaf senescence.

### Gene Ontology Enrichment

Gene ontology (GO) analysis found that the DEGs in C1 were significantly enriched (*p*-value < 0.01) in 76 GO terms (Supplementary Table S7), including 36 terms on the biological process (BP), six on cellular component (CC), and 34 on molecular function (MF). In terms of the BP terms, the upregulated DEGs were enriched in the GO terms of the regulation of metabolic and biosynthetic processes (Supplementary Figure S1), while the downregulated DEGs were enriched in the GO terms of metabolic and biosynthetic regulation processes, ammonium transport, nitrate metabolic process, oxidation–reduction process, and so on (Supplementary Figure S2). In C2, 114 GO terms were significantly enriched (*p*-value < 0.01) with DEGs (Supplementary Table S8), including 61 on BP, 10 on CC, and 43 on MF, respectively. Among the BP terms, the downregulated DEGs in C2 comparison were enriched in the regulation of the metabolic process, regulation of biosynthetic process, and photosynthesis (Supplementary Figure S3). The DEGs in C3 were enriched (*p*-value < 0.01) in 132 GO terms (Supplementary Table S9) including 59 terms on BP, 10 on CC, and 63 on MF, respectively. Among the BP terms, the upregulated DEGs were enriched in the GO terms of the hormone pathway (Supplementary Figure S4), while the downregulated DEGs were involved in the oxidation–reduction process, photosynthesis, and generation of precursor metabolites, energy, and carbon fixation (Supplementary Figure S5). The DEGs in C4 were enriched (*p*-value < 0.01) in 152 GO terms (Supplementary Table S10), including 61 terms on BP, 20 on CC and 71 on MF, respectively. Among them, the upregulated DEGs were enriched in the GO terms of the hormone pathway, oxidation–reduction, and carbohydrate metabolic processes (Supplementary Figure S6), while the downregulated DEGs were mostly related to the regulation of metabolic and biosynthetic processes (Supplementary Figure S7).

**FIGURE 5:**
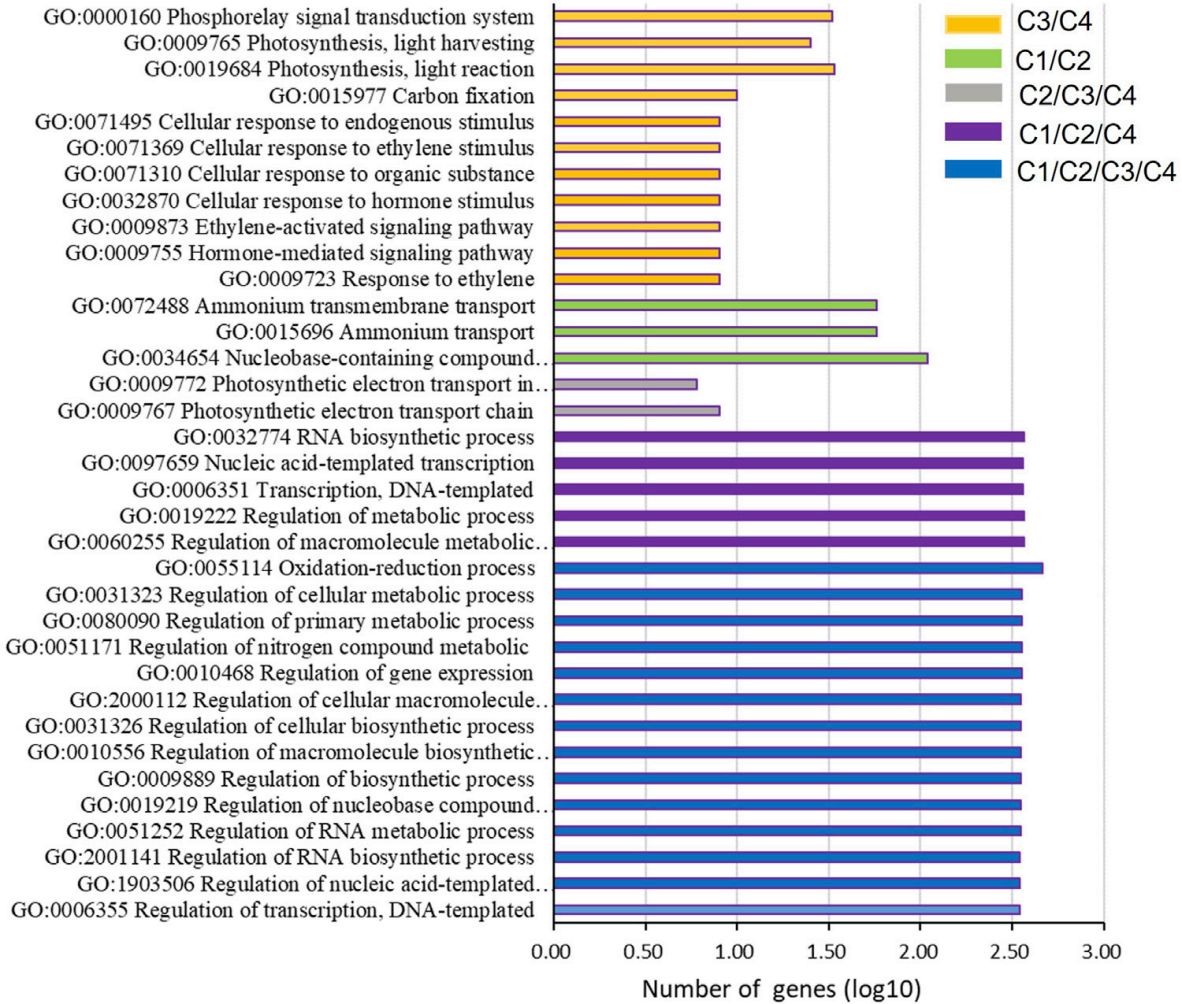
GO terms on the biological process overlapping in at least two of the four pairs of comparison.

To explore the regulation mechanism of gene expression during the leaf senescence process, the common BP GO terms (*p*-value < 0.001) were analyzed among the four pair comparisons (Figure 5). Interestingly, 14 common GO terms were found in all comparisons (C1, C2, C3, and C4), these GO terms were related to the regulation of transcription (GO:0006355 and GO:1903506), biosynthetic process (GO:2001141, GO:0009889, GO:0010556, GO:0031326 and GO:2000112), metabolic process (GO:0051252, GO:0019219, GO:0080090, and GO:0031323), gene expression (GO:0010468), nitrogen compound metabolic (GO:0051171), and oxidation-reduction process (GO:0055114). Moreover, two common GO terms only existed among C2, C3, and C4, including the photosynthetic electron transport chain (GO:0009767) and photosynthetic electron transport in photosystem II (GO:0009772), but not in C1. In addition, there were three common GO terms between C1 and C2, including nucleobase-containing compound biosynthetic (GO:0034654), ammonium transport (GO:0015696), and ammonium transmembrane transport (GO:0072488). There were 11 common GO terms between C3 and C4, and these GO terms mainly involved in senescence, including the hormone pathway (GO:0009723, GO:0009755, GO:0009873, GO:0032870, and GO:0071369), photosynthesis pathway (GO:0019684, GO:0009765 GO:0000160), carbon fixation (GO:0015977), cellular response to organic substance (GO:0071310), and endogenous stimulus (GO:0071495).

In all, numerous genes related to the biosynthetic and metabolic processes, especially the nitrogen metabolic pathways, were closely related to the whole leaf senescence process (M1∼M5). In the early stage (M1∼M3), genes involved in the biosynthetic and ammonium transport pathways were downregulated with the increase in leaf senescence. At the later senescence (M4 and M5), with the upregulated of hormone and the degradation of chlorophyll, the activity of photosynthesis, ammonium transport, and nitrogen metabolic decreased.

### Cluster of Expression Patterns of DEGs

**FIGURE 6:**
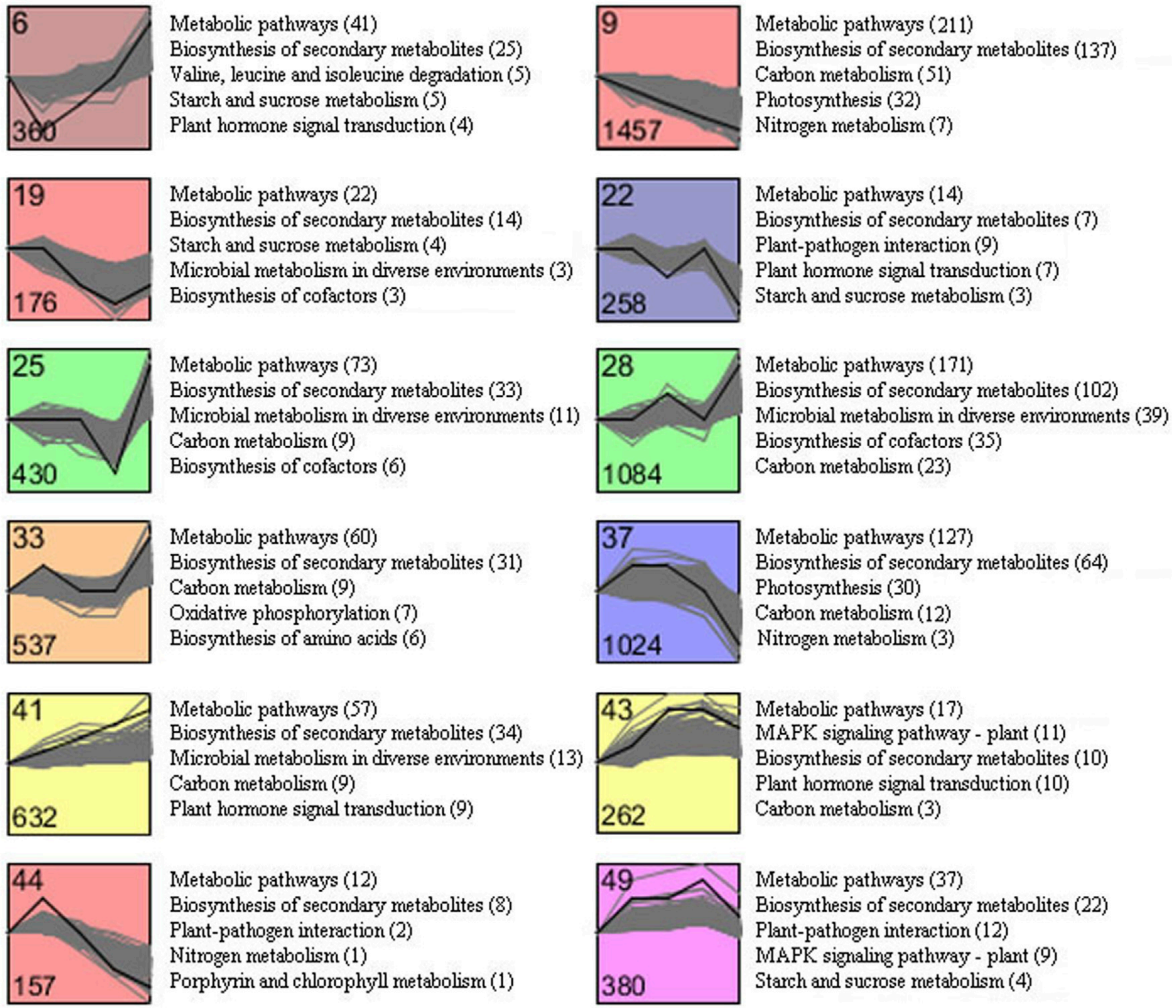
Cluster analysis of DEGs with significant expression profile changes and KEGG pathway enrichment analysis.

To explore the time course differential expression profile of DEGs identified from C1 to C4, the STEM analysis for all DEGs was performed. The results of the STEM analysis showed that these DEGs were significantly enriched in 12 profiles (Figure 6). The gene expression trend of clustering in each profile was similar. Among them, profile nine contained the largest gene set, with 1,457 genes, followed by profile 28 (1,084 genes) and profile 37 (1,024 genes), respectively. In addition, the expression of genes included in profile nine was continuously downregulated during tobacco senescence. In contrast, 632 genes included in profile 41 were continuously upregulated. An overview of the KEGG pathway enrichment also exhibited a global description of the enriched pathways in each profile with similar expression pattern. The result showed that 12 clusters were mainly enriched in the “biosynthesis of secondary metabolites,” “plant hormone signal transduction,” “carbon metabolism,” “starch and sucrose metabolism,” “nitrogen metabolism,” and “photosynthesis.” Notably, “carbon metabolism,” which related to biochemical substance transport during the senescence process, was enriched in the majority clusters (profile 9/25/28/33/37/41/43), and “starch and sucrose metabolism” was enriched in clusters 6, 19, 22, and 49, and the “nitrogen metabolism” pathway was enriched in clusters 9, 37, and 44, while the “photosynthesis” pathway was enriched in clusters 9 and 37.

### Transcriptional Modules Related to Leaf Senescence

**FIGURE 7:**
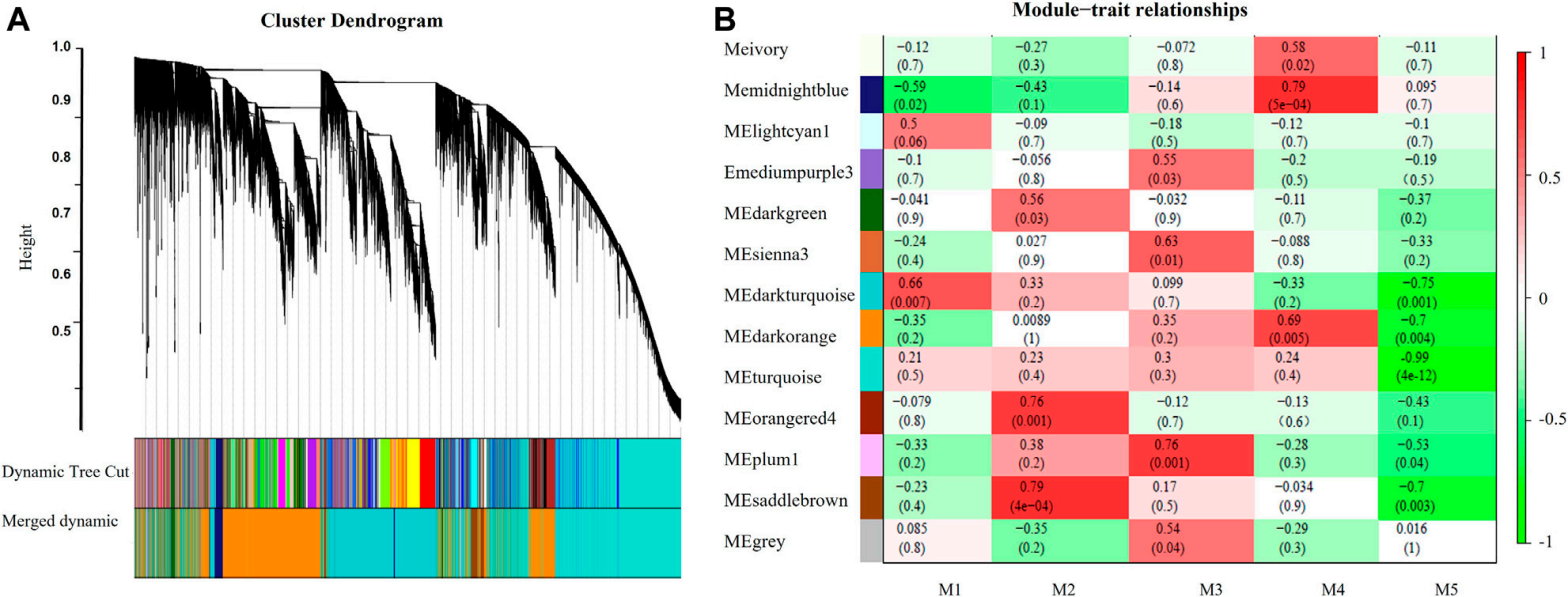
Network analysis dendrogram showing modules identified by the WGCNA. **(A)** Hierarchical cluster tree. **(B)** Module-trait relationships of modules significantly correlated with leaf senescence. Each cell contains the corresponding correlation and *p*-value.

After modules with similar expression profiles were merged, a total of 13 gene modules were identified. Association analysis was performed to detect between modules and five leaf senescence stages (Figure 7). Module–trait association analysis found that five modules were significantly correlated with a specific senescence stage (with the cutoffs, *r* > 0.75 and *p* value <0.01). The midnightblue module showed positive correlation with the M4 stage (*r* = 0.79, *p* = 5e-04). The turquoise module showed strong negative correlation with the M5 stage (*r* = -0.99, *p* = 4e-12). The module of orangered4 (*r* = 0.76, *p* = 0.001) and saddlebrown (*r* = 0.79, *p* = 4e-04) were both positively associated with the M2 stage. The plum1 module showed positive correlation with the M3 stage (*r* = 0.76, *p* = 0.001). Three modules (turquoise, midnightblue, and saddlebrown) with top correlation values were selected for further co-expression networks analysis. To identify the candidate hub genes, the top ten connected genes in each selected module were ranked by the using Maximal Clique Centrality (MCC) method with Cytoscape software (Table 1). In the positively related midnightblue and saddlebrown modules, the majority connected genes were involved in signaling transduction, hormone metabolism, and stress response, such as lipoxygenase, redox thioredoxin, zinc finger protein, kinase, ethylene receptor, chitin-inducible gibberellin-responsive protein 1, calmodulin (CAM)-binding protein, signaling G-proteins, development, and cell death protein. In the negatively related turquoise modules, the majority connected genes were related to protein degradation, hormone metabolism, and secondary metabolism, such as ribosomal protein, serine carboxypeptidase–like 25, P-loop containing nucleoside triphosphate hydrolase protein, adenine nucleotide alpha hydrolase–like protein, and so on.

**TABLE 1:** Candidate hub genes screened from specific modules related to leaf senescence.

Gene ID	Annotation information	Module
Nitab4.5_0007252g0020	Lipoxygenase, oxidoreductase activity	Midnightblue
Nitab4.5_0004905g0080	B12D protein	Midnightblue
Nitab4.5_0003118g0010	Redox thioredoxin	Midnightblue
Nitab4.5_0000003g0900	Zinc finger protein, TAZ-type	Midnightblue
Nitab4.5_0001492g0080	Protein kinase	Midnightblue
Nitab4.5_0000622g0230	B-cell receptor–associated 31–like, intracellular protein transport	Midnightblue
Nitab4.5_0004928g0060	Ethylene receptor, hormone metabolism, signal transduction	Midnightblue
Nitab4.5_0001032g0040	Zinc finger transcription factor	Midnightblue
Nitab4.5_0000029g0320	Phosphoglycerate kinase	Midnightblue
Nitab4.5_0003436g0140	Glucan synthase–like, cell wall component callose synthesis	Midnightblue
Nitab4.5_0000749g0090	Chitin-inducible gibberellin-responsive protein 1	Saddlebrown
Nitab4.5_0005796g0010	SUMO activation enzyme	Saddlebrown
Nitab4.5_0000062g0280	Calmodulin (CAM)-binding protein	Saddlebrown
Nitab4.5_0001144g0030	Putative GTP-binding protein, signaling G-proteins	Saddlebrown
Nitab4.5_0005528g0110	Protein phosphatase 2C protein	Saddlebrown
Nitab4.5_0000105g0190	Trichome birefringence–like (TBL) gene	Saddlebrown
Nitab4.5_0002487g0010	Protein BYPASS-related, alpha crystallin/Hsp20 domain	Saddlebrown
Nitab4.5_0000859g0210	Unknown protein, located in chloroplast inner membrane	Saddlebrown
Nitab4.5_0002966g0050	O-fucosyltransferase protein	Saddlebrown
Nitab4.5_0002231g0050	Development and cell death (DCD) protein	Saddlebrown
Nitab4.5_0006967g0050	P-loop containing nucleoside triphosphate hydrolases protein, sulfotransferase activity	Turquoise
Nitab4.5_0004921g0010	Peroxin 11 (PEX11) protein	Turquoise
Nitab4.5_0001951g0050	Ribosomal protein L36e protein	Turquoise
Nitab4.5_0003525g0050	Adenine nucleotide alpha hydrolase–like superfamily protein	Turquoise
Nitab4.5_0001195g0030	Polynucleotidyl transferase, ribonuclease H–like protein	Turquoise
Nitab4.5_0001663g0280	G2-like transcription factor	Turquoise
Nitab4.5_0009379g0010	Serine carboxypeptidase–like 25	Turquoise
Nitab4.5_0014465g0010	Ring-box 1–like protein, component of the SCF ubiquitinization complex mediating auxin responses	Turquoise
Nitab4.5_0002017g0010	Haloacid dehalogenase–like hydrolase (HAD) protein	Turquoise
Nitab4.5_0002334g0060	Leucine-rich repeat protein	Turquoise

### DEG Validation by qRT-PCR

**FIGURE 8:**
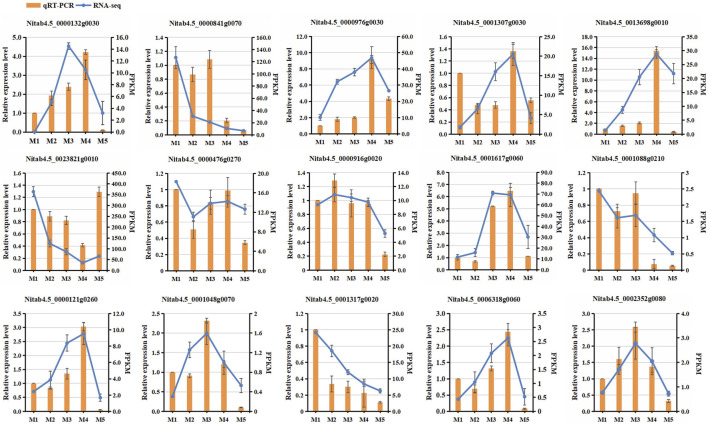
Comparison of expression levels of 15 selected genes between RNA-seq and qRT-PCR analyses at five maturity stages.

To validate the RNA-seq data, the expression patterns of fifteen differentially expressed genes (DEGs) in the five maturity stages were selected for qRT-PCR (Supplementary Table S1). The results showed that the expression trends of these fifteen genes were consistent with those examined by RNA-seq (Figure 8), confirming the reliability of the RNA-seq data.

## Discussion

Leaf senescence is a complex process that plays an important role in regulating nutrient distribution and improving plant adaptability to the environment. During the process of leaf senescence, a series of nutrients are redistributed and mainly focus on the degradation of biological macromolecules such as proteins, lipids, nucleic acids, and nutrients released from cell catabolism. These macromolecules are transported to young organs such as new buds, young leaves, developing fruits, and flowers as their energy source (carbon source and nitrogen source), or to be stored in trunks for next growing season (Guo and Gan, 2005). Usually, nitrogen is transported as the form of glutamine to young tissues to complete the nitrogen recycling (Guo et al., 2004), and most of the nitrogen in corn, rice, and wheat grains originates from the recycling of nitrogen, which has a significant impact on crop yield (Gregersen et al., 2008). In this study, the content of nitrogen was decreased with the progress of maturity, and the result also indicated that the nitrogen was transported from source leaves to sink organs during the process of tobacco leaf senescence. In addition, the carbohydrates are the main substance changed in the process of leaf senescence, especially sugars. There are two hypotheses about sugar induced plant senescence: sugar accumulation and sugar starvation. Studies have shown that the accumulation of sugar in cells accelerates or induces leaf senescence (Pourtau et al., 2006; Wingler et al., 2006). In contrast, some studies found that dark treatment causes sugar starvation in leaves and induces senescence. Exogenous sugar treatment could delay leaf senescence (Fujiki et al., 2001). Moreover, it was reported that leaf senescence is driven by a series of genes senescence-associated genes (SAGs) (Gan and Amasino, 1997), and sugar accumulation could induce the expression of senescence-associated genes (SAGs) in the early stage of senescence, whereas the expression of SAGs would be inhibited by the sugar in the later stage of senescence (Paul and Pellny, 2003). In this study, contents of total sugar and reducing sugar were increased first and then dropped with increasing maturity. Obviously, sugar accumulation appeared in the early stages (M1∼M3) of tobacco leaf senescence and then decreased with the acceleration of senescence (M4 and M5). Our result was in consistent with the previous study (Masclaux et al., 2000). Furthermore, numerous DEGs were detected based on the transcriptomic analysis at five senescence stages. The GO enrichment analysis showed that these DEGs were enriched in various enzyme activities associated carbohydrate metabolism, nitrogen metabolism, ammonia transport, and photosynthesis. Especially, the upregulated DEGs in C4 were enriched in the GO terms of the hormone pathway, oxidation–reduction, and carbohydrate metabolic processes. Our result indicated the changes of sugar content at different senescence stages may closely be related to leaf senescence and the sugar accumulation in the early stage of senescence (M1∼M3). The decline in the sugar content at the late senescence stage may all be induced or increased the expression of a series SAGs that lead to senescence. With the decreasing photosynthetic rate and the activities of various enzymes involved in carbohydrate synthesis, the sugar transport from source leaves to sink organs leads to sugar starvation stress of leaf and finally accelerates leaf senescence.

Generally, photosynthetic capacity of leaves will decrease with the reduction of chlorophyll. In this study, the chlorophyll content showed a significant declining tendency with the increasing degree of maturity from M1 to M5. While the DEGs related to photosynthesis in profiles 9 and 37 mostly showed downregulation according to STEM and KEGG analyses, the result indicated that the content of chlorophyll and the expression level of DEGs involved in photosynthesis has a positive correlation. It has been reported that the expression of some key genes involved in the carbon metabolism, starch and sucrose metabolism, and nitrogen metabolism is positively correlated with metabolite accumulation such as sugar, starch, and nicotine (Li et al., 2017). In our study, DEGs related to the carbon metabolism, starch and sucrose metabolism, and nitrogen metabolism were found to be clustered in different expression patterns (Figure 6). It is obvious that it could not establish direct correlation between the metabolites accumulation and the expression of genes based on the whole transcriptome inferring that the synthesis of metabolic substances could be complicated with that of the co-expressed genes.

Hormones play important roles in the plant senescence. It is reported that cytokinin (CTK) and auxin (IAA) inhibit leaf senescence, while ethylene and gibberellin (GA) promote leaf senescence (Jibran et al., 2013; Schippers et al., 2015). The ethylene content is gradually growing with the increase in ethylene synthase activity during leaf senescence, so that it is one of the significant signs of leaf senescence (Lim et al., 2007). Oh et al. (1997) found ethylene mutant *ein2* can delay senescence in *Arabidopsis*, while ethylene-insensitive mutants such as *etr1*, *etr2*, *ein1*, *ein3*, and *ein4* could accelerate senescence (Hua et al., 1998). In this study, DEGs of late senescence stages were enriched in hormone-mediated pathways, especially in the ethylene pathway (Supplementary Figures S4, S6), and these genes may play important roles in tobacco leaf senescence. Moreover, the core gene *Nitab4.5_0004928g0060* screened by the co-expression network analysis in this study (Table 1) was the homologous of *AT3G04580.1*(*ein4*), which is involved in the ethylene activation signal pathway in *Arabidopsis*. The results indicated that *Nitab4.5_0004928g0060* may also play roles in the process of tobacco senescence according to the similar pathway.

In the process of leaf senescence, there are dramatic changes at the physiological and molecular levels, including photosynthesis declining and organelles degrading accompany with a great quantity of reactive oxygen (ROS) generated by redox reactions, which makes the senescent leaves in adversity. A large amount of ROS accumulated in cells accelerates the degradation of the biofilm system by peroxidation and damage of cellular organelles, followed by the yellowing and senescence of leaves (Apel and Hirt, 2004). It was reported that inhibiting H_2_O_2_ (a common ROS) could delay leaf senescence in *Arabidopsis thaliana* (Bieker et al., 2012). In this study, one peroxidase gene that is related to the production and scavenging of ROS was identified as the hub gene (*Nitab4.5_0004921g0010*) based on the WGCNA. This gene was upregulated during the process of leaf senescence, which confirmed the accumulation of ROS and closely associated with tobacco leaves senescence.

It was reported that stress response pathways appear to be involved in the onset of senescence (Guo and Gan, 2005), and many of the defense response (DR) genes were identified as SAGs (Quirino et al., 2000; Buchanan-Wollaston et al., 2005). Previous studies have reported that salicylic acid (SA) and jasmonic acid (JA) are positive regulators for defense against pathogens and senescence-induced metabolisms (Morris et al., 2000; Lim et al., 2007; Wasternack, 2007). Moreover, abscisic acid (ABA) has been proved to be indispensable in regulating plant senescence and responding to biotic and abiotic stresses, and external application of ABA could increase the expression of SAGs (Lim et al., 2007; Choi and Hwang, 2011). In addition, MYC2, a member of bHLH TFs, can bind to the promoter of *SAG29* to activate the JA-mediated senescence pathway (Qi et al., 2015). In this study, the WGCNA showed three modules, namely, saddlebrown, midnightblue, and turquoise, which were in high correlation with M2, M4, and M5 stages, respectively. MapMan analysis found that numerous DEGs (Compare to M1) included in these modules were related to stress response, such as hormone (IAA, GA, ethylene, JA, and SA), transcription factors (WRKY, bZIP, MYB, ERF, and DOF), defense gene, cell wall, heat shock proteins, proteolysis, and secondary metabolites (Supplementary Table S11). Furthermore, majority of these DEGs were upregulated. A common factor related to various stress responses and senescence is increased levels of ROS (Zimmermann et al., 2006; Leng et al., 2017), which may induce the upregulation of the same group of genes during senescence and in response to stress treatments. Our result indicated that a wide overlap exists between senescence and stress responses (Chen et al., 2002), and leaf senescence is jointly regulated by multiple factors, including a variety of cascade amplification effects and signal transduction.

## Conclusion

Leaf senescence is a complex process involving many molecular events along with physiological and biochemical changes in plants. In this study, significant dynamic changes in nitrogen, sugars, and the DEGs related to metabolites accumulation were identified according to the analyses of biochemical properties and transcriptome at five maturity stages (M1∼M5) of tobacco leaves. GO analysis found that the DEGs were enriched in the biosynthetic, metabolic, photosynthesis, and redox processes, especially the nitrogen metabolic pathways that were closely related to the whole leaf senescence process. The DEGs related to the carbon metabolism, starch and sucrose metabolism, nitrogen metabolism, and photosynthesis showed distinct expression patterns. A total of 30 core genes were examined by Weight Gene Co-expression Network Analysis (WGCNA), and these genes appeared to have an important role in the regulatory of tobacco leaf senescence. Our results have provided useful information for future functional investigation of leaf senescence in plants.

## Data Availability

The datasets presented in this study can be found in online repositories. The names of the repository/repositories and accession number(s) can be found below: https://www.ncbi.nlm.nih.gov/sra/PRJNA772550.
