# Supplementary material for: Integrative Analyses of Biochemical Properties and Transcriptome Reveal the Dynamic Changes in Leaf Senescence of Tobacco (Nicotiana tabacum L.)
Source: Front Genet. 2021 Dec 22;12:790167. doi: 10.3389/fgene.2021.790167 (PMC8727547; doi:10.3389/fgene.2021.790167)
Supplement: Supplementary file 1 [file DataSheet1.ZIP › Supplemental tables and figures/Table S1.docx]

| Primer Name | Forward Primer (5′→3′) Sequence | Reverse Primer (5′→3′) Sequence |
| --- | --- | --- |
| NtActin | ACCTCTATGGCAACATTGTGCTCAG | CTGGGAGCCAAAGCGGTGATT |
| Nitab4.5_0000132g0030 | CTCATTATGCGTCGCAATTC | ATTCTTCAGGTTGCCTTTGC |
| Nitab4.5_0000841g0070 | CCTCACAAACACCACCTACG | CAAGCACGTTCGTAAGCATT |
| Nitab4.5_0000976g0030 | CCGAGGGTCTATCGAAAACA | TTGCAGAACAGAAGGCAATG |
| Nitab4.5_0001307g0030 | GGCAGGGTAAACAGGTTGAA | GATGGTGCCAGAGAACTGGT |
| Nitab4.5_0013698g0010 | CATGGAGGTGGAACTGCTTT | GGGCCTTGACTGTCCAATAA |
| Nitab4.5_0023821g0010 | GCACTGATTGCACTGAGGAA | AAGATCCATGGACGGAGTTG |
| Nitab4.5_0000476g0270 | GCGCATTGAGGCATTGAAGA | AGCTTTTCCAGTTCCCTCCT |
| Nitab4.5_0000916g0020 | GTGCCAAAGATCTGCCAACA | TTGGTTTAAGCGATTCGGGC |
| Nitab4.5_0001617g0060 | AGGTTGGGTACTGCCAACAG | GGGGTAACTTTGAGGGTGGT |
| Nitab4.5_0001088g0210 | TGCTTGCTGTGTCATCTTTGA | TGGTAGCAAATCTCCGTCGT |
| Nitab4.5_0000121g0260 | CCAACACCAGAGGGGTTAGA | CCCTCTTGGGAAAAACCATT |
| Nitab4.5_0001048g0070 | AACATGGTCCTGGAAACTGG | CCAAGAAGAGCTTGGAGGTG |
| Nitab4.5_0001317g0020 | GGGGTGAGAGTGATGAAGCT | AAACTGCCGCATTTGACCAT |
| Nitab4.5_0006318g0060 | GGACCATGGACTCCTGAAGA | TCTTGCTACATCTGCGCAAC |
| Nitab4.5_0002352g0080 | AGGACCATGGACACCAGAAG | TCCACGTTTAATCCCTGGAC |

**Table S1.** Gene-specific primers used for qRT-PCR analysis.
