# Supplementary material for: Integrative Analyses of Biochemical Properties and Transcriptome Reveal the Dynamic Changes in Leaf Senescence of Tobacco (Nicotiana tabacum L.)
Source: Front Genet. 2021 Dec 22;12:790167. doi: 10.3389/fgene.2021.790167 (PMC8727547; doi:10.3389/fgene.2021.790167)
Supplement: Supplementary file 1 [file DataSheet1.ZIP › Supplemental tables and figures/Table S2.docx]

**Table S2.** Statistics of RNA-seq reads

| Samples | Clean reads | Uniquely mapped reads  (%) | Multiply mapped reads  (%) |
| --- | --- | --- | --- |
| M1-1 | 47,946,038 | 41,018,104 (85.55%) | 4,646,684 (9.69%) |
| M1-2 | 54,452,730 | 46,299,985 (85.03%) | 5,688,575 (10.45%) |
| M1-3 | 56,755,856 | 48,368,804 (85.22%) | 5,643,425 (9.94%) |
| M2-1 | 62,421,104 | 51,851,356 (83.07%) | 7,379,679 (11.82%) |
| M2-2 | 51,199,464 | 43,845,721 (85.64%) | 5,024,538 (9.81%) |
| M2-3 | 51,215,348 | 44,045,750 (86.00%) | 4,739,727 (9.25%) |
| M3-1 | 47,338,570 | 40,782,734 (86.15%) | 4,247,944 (8.97%) |
| M3-2 | 55,051,766 | 47,464,655 (86.22%) | 4,816,950 (8.75%) |
| M3-3 | 50,673,968 | 43,970,819 (86.77%) | 4,033,358 (7.96%) |
| M4-1 | 48,894,700 | 42,191,109 (86.29%) | 4,309,974 (8.81%) |
| M4-2 | 45,307,044 | 39,225,342 (86.58%) | 3,862,292 (8.52%) |
| M4-3 | 52,086,882 | 45,068,609 (86.53%) | 4,457,845 (8.56%) |
| M5-1 | 50,623,950 | 45,061,933 (89.01%) | 3,291,078 (6.50%) |
| M5-2 | 51,008,172 | 45,114,538 (88.45%) | 3,636,715 (7.13%) |
| M5-3 | 54,106,026 | 47,826,519 (88.39%) | 3,536,544 (6.54%) |

Note: Reads were mapped to the tobacco reference genome (Edwards, 2017 version).
